# The complete plastid genome of *Jeffersonia diphylla* and its phylogenetic position inference

**DOI:** 10.1080/23802359.2019.1696250

**Published:** 2019-12-09

**Authors:** Rui Li, Meifang Dong

**Affiliations:** a Food Inspection and Testing Institute of Henan Province, Zhengzhou, China;; b Key Laboratory of Plant Stress Biology, School of Life Sciences, Henan University, Kaifeng, China

**Keywords:** Genome skimming, *Jeffersonia diphylla*, phylogenetic position

## Abstract

*Jeffersonia diphylla*, which belongs to Berberidaceae, is a perennial herb native to North America. In the present study, we determined the first plastome of *J. diphylla* using genome skimming approach. The pastome of *J. diphylla* is 152,842 bp in length, with a large single copy region (LSC) of 82,266 bp and a small single copy region (SSC) of 19,284 bp separated by a pair of inverted repeats (IRs) of 25,646 bp. It encodes 113 unique genes, consisting of 79 protein-coding genes, 30 tRNA genes, and four rRNA genes, with 19 duplicated genes in the IR regions. Phylogenetic analysis indicates that *J. diphylla* is sister to *Plagiorhegma dubium*, subsequently is sister to a clade including *Diphylleia*, *Sinopodophyllum*, *Podophyllum*, *Dysosma*, *Achlys*, and *Epimedium*.

*Jeffersonia diphylla* (L.) Pers. (Berberidaceae) is an herbaceous perennial of rich, usually calcareous, forests in eastern North America (Baskin and Baskin 1989). Its natural distribution extends from southern Ontario and New York to Wisconsin and northeastern Iowa south to Maryland and Alabama (Gleason 1968). The plants of *J. diphylla* were used medicinally by Native Americans for treatment of dropsy, gravel, and urinary ailments, and in poultices for sores and ulcers (Moerman 1988). However, the phylogenetic position of *J. diphylla* was still unclear, and the studies regarding to it, especially for genetic resource, are scarce. In this study, the plastome of *J. diphylla* was firstly determined using genome skimming data. The genome sequence was registered into GenBank with the accession number MN648410.

Fresh leaves of *J. diphylla* were collected from the Clifty Falls State Park (United States; 85°25′07.32″W, 38°58′04.62″N) and a voucher specimen (*Pan Li; LP162037*) was deposited at the Herbarium of Zhejiang University (HZU). Total genomic DNA was extracted from silica-dried leaf tissue using Plant DNAzol Reagent (LifeFeng, Shanghai) according to the manufacturer’s protocol. The high-quality DNA was sheared and the paired-end library was prepared and sequenced on an Illumina HiSeq X10 at Beijing Genomics Institute (BGI, Wuhan, China). The raw Illumina reads were quality trimmed (Phred score < 30) and assembled into contigs using the CLC Genomics Workbench (CLC Inc. Aarhus, Denmark) to reconstruct the plastome with *Plagiorhegma dubium* (GenBank accession number: NC_038103) as a reference. The plastome was annotated using the software Geneious R11 (Biomatters, Auckland, New Zealand) following description in Liu et al. (2017) and Liu et al. (2018), and a graphical map of the annotated circular plastome was generated using the OGDRAW program (Lohse et al. 2013). Phylogenetic position of *J. diphylla* was inferred using the whole plastome sequences, and maximum-likelihood (ML) was implemented in RAxML-HPC v8.1.11 on the CIPRES cluster (Miller et al. 2010).

The plastome of *J. diphylla* is 152,842 bp in length and had a typical quadripartite structure comprising two copies of IRs (25,646 bp) separated by the LSC (82,266 bp) and SSC (19,284 bp) regions. The plastome contains 113 unique genes including 79 protein-coding genes, 30 tRNA genes, and four ribosomal RNA genes, with 19 duplicated in the IR regions. Six tRNA genes and eight protein-coding genes contained single intron, and three genes including *rps*12, *clp*P, and *ycf*3 contained two introns. The overall GC content of the total length, LSC, SSC, and IR regions is 38.1%, 36.4%, 31.9%, and 43.3%, respectively. The phylogeny revealed that *J. diphylla* and *P. dubium* formed a clade with high support, which is sister to *Diphylleia*, *Sinopodophyllum*, *Podophyllum*, *Dysosma*, *Achlys*, and *Epimedium* (Figure 1).

**Figure 1.**
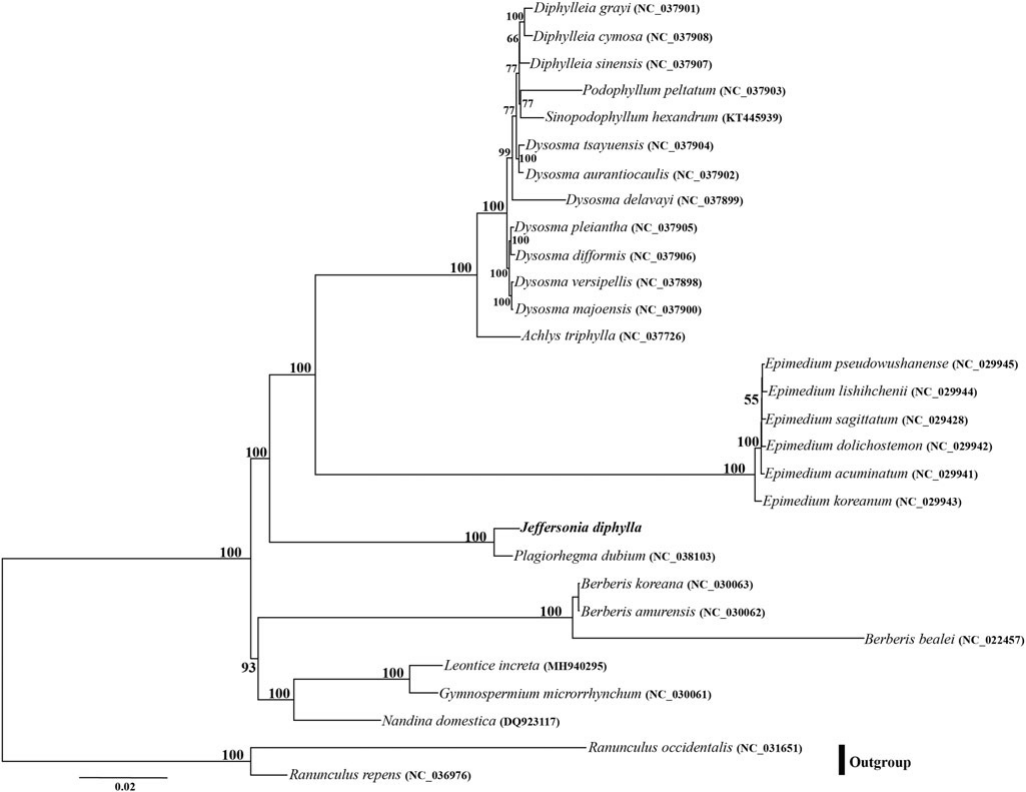
Phylogenetic position of *Jeffersonia diphylla* using maximum-likelihood (ML) based on whole chloroplast genome sequences. Numbers above the lines represent ML bootstrap values. ML bootstrap <50% was not shown.
